# Precision Food Safety: a Paradigm Shift in Detection and Control of Foodborne Pathogens

**DOI:** 10.1128/mSystems.00164-19

**Published:** 2019-06-11

**Authors:** Jasna Kovac

**Affiliations:** a Department of Food Science, The Pennsylvania State University, University Park, Pennsylvania, USA

**Keywords:** precision food safety, food supply chain, genomics, metagenomics, microbiomes

## Abstract

The implementation of whole-genome sequencing in food safety has revolutionized foodborne pathogen tracking and outbreak investigations. The vast amounts of genomic data that are being produced through ongoing surveillance efforts continue advancing our understanding of pathogen diversity and genome biology. Produced genomic data are also supporting the use of metagenomics and metatranscriptomics for detection and functional characterization of microbiological hazards in foods and food processing environments. In addition to that, many studies have shown that metabolic and pathogenic potential, antimicrobial resistance, and other phenotypes relevant to food safety can be predicted from whole-genome sequences, omitting the need for multiple laboratory tests. Nevertheless, further work in the area of functional inference is necessary to enable accurate interpretation of functional information inferred from genomic and metagenomic data, as well as real-time detection and tracking of high-risk pathogen subtypes and microbiomes.

## Abstract (precis)

The implementation of whole-genome sequencing in food safety has revolutionized foodborne pathogen tracking and outbreak investigations. The vast amounts of genomic data that are being produced through ongoing surveillance efforts continue advancing our understanding of pathogen diversity and genome biology.

## PERSPECTIVE

Microbiological food safety has traditionally been monitored using culture-based phenotypic methods for detection, characterization, and identification of target foodborne pathogens. Pathogens have primarily been studied at a species level and out of the context of microbial communities in which they reside. The breakthrough in high-throughput sequencing in the 2000s enabled the development of precise subtyping methods for tracking of foodborne pathogens and microbial communities. The implementation of these methods is profoundly changing foodborne pathogen surveillance and is expected to increasingly inform the control of foodborne pathogens in the coming years.

I discuss three areas of opportunities that have tremendous potential for a paradigm shift in the detection and control of foodborne pathogens. The primary area pertains to the definition of a foodborne pathogen and associated ramifications for public health and the food industries. The next area considers opportunities for surpassing whole-genome sequencing to detect unknown pathogens, and the third area highlights the opportunities for improved control of foodborne pathogens through informed manipulation of food processing environmental microbiomes.

## FOODBORNE PATHOGENS: NOT ALL SUBTYPES ARE CREATED EQUAL

Foodborne pathogens have traditionally been mostly defined and regulated at a species level. The current “zero-tolerance” regulation for certain pathogen species reflects such notions. These regulations result in increased food waste and costs for the food industry and consumers, without necessarily achieving the desired public health outcome. Subtypes of pathogen species vary in their ability to cause disease in different hosts. For example, some serotypes of *Salmonella* are highly risky for humans, while others typically cause the disease in animals and are far less frequently reported to cause illness in humans (1). Whether all should be treated and regulated equally is debatable and depends on the level of risk that is deemed acceptable.

Furthermore, it is becoming increasingly evident that not all members of the foodborne pathogen species groups such as the Bacillus cereus and Clostridium botulinum groups have equal capacities to cause foodborne illness and have detrimental effects on human cells (2, 3). In some cases, the pathogenic potential may be predicted based on the presence of virulence genes or phylogenetic clustering, while in other cases predictions are more challenging. We developed a bioinformatics tool, BTyper, to facilitate sequence-based characterization of the B. cereus group isolates and aid in characterization of the pathogenic potential of distinct isolates (2, 4). Currently, available data on pathogenicity of subtypes are limiting our ability to predict the pathogenic potential of individual strains with sufficient accuracy; hence, the industry and regulation are erring on the side of caution, and rightly so. Nevertheless, this comes at a considerable cost for the industry and highlights the need for improved food safety diagnostics. For example, Fonterra, one of the world's largest dairy exporters, suffered immense economic damage after the 2013 incident that involved misclassification of a foodborne nonpathogenic *Clostridium* isolate as deadly Clostridium botulinum. This example illustrates the need for availability of accurate pathogenicity prediction tools in food industry. I anticipate that collaborative research characterizing the pathogenic potential of distinct subtypes within certain pathogen species and species groups will allow for quantitative risk assessment on a pathogen subtype level in the next 5 to 10 years. The outcomes of these efforts are likely going to have a significant positive impact on public health, food security, and economic viability of food industries if implemented correctly.

## FOODBORNE PATHOGEN DETECTION: TAKING A STEP BEYOND WGS

The implementation of whole-genome sequencing (WGS) in foodborne pathogen surveillance in the United States resulted in a rapid increase in public availability of foodborne pathogen whole-genome sequences (5). By the end of the first quarter of 2019, GenomeTrakr and PulseNet networks have sequenced over 317,000 foodborne pathogen genomes, according to the fact sheet published on the GenomeTrakr website (https://www.fda.gov/Food/FoodScienceResearch/WholeGenomeSequencingProgramWGS/ucm403550.htm). The positive impacts of the real-time WGS surveillance are evident from the outcomes of the Listeria monocytogenes surveillance program that resulted in more frequently detected but smaller outbreaks since the implementation of the WGS in routine pathogen surveillance (6). The critical component of successful implementation of such enhanced surveillance programs is national and international data sharing and methods standardization. The data analyses and storage are supported by the National Center for Biotechnology Information, and the latter is currently coordinated by the Genomics for Food Safety (Gen-FS) within the United States and by the Global Microbial Identifier at international scale (5). Global standardization still represents a significant challenge and is a work in progress, while the positive impacts of the increased global availability of WGS are already evident. One of many positive outcomes is a successful investigation of a *Salmonella* Bareilly outbreak where comparative genomics led to the identification of an international source of contaminated tuna that would have otherwise remained under the radar (7). Apart from high-profile foodborne pathogens, WGS monitoring is proving to be relevant also for the investigation of underresearched pathogens such as the Bacillus cereus group studied in my lab (8). Using whole-genome sequencing in conjunction with an epidemiological investigation and phenotypic characterization of outbreak-associated isolates, we identified Bacillus paranthracis, a recently discovered B. cereus group species, as a causative agent of the outbreak (8). Using traditional methods, this pathogen species would not have been identified, as phenotypic methods lack sufficient discriminatory power.

Despite outstanding successes of WGS in food safety, microbial isolation workflows coupled with WGS still overlook an estimated 38.4 million cases of domestically acquired foodborne illnesses in the United States that are caused by unspecified agents (9). Unidentified agents cause an astonishing 80% of all estimated cases of foodborne illnesses occurring in the United States on a yearly basis (9). I argue that there is a need for addressing this gap in knowledge by piloting a metagenome-based screening of the food supply chain and clinical samples. The advanced machine learning methods that have been successfully implemented in source attribution of pathogens can be implemented for the detection of novel pathogens as well (10, 11).

Successful implementation of genomics in food safety paved the path to probing the utility of metagenomic sequencing in characterization of microbiomes in the food supply chain. Shotgun metagenomic sequencing has been successfully used to detect Escherichia coli and *Salmonella* at a low contamination level and with a strain-level resolution directly in foods, without isolation (12, 13). However, with improved sensitivity, the challenge of differentiation between live and dead cells of pathogens becomes more pronounced. This can be overcome using preenrichment steps following quasimetagenomics workflows (13). Large sequencing efforts by government agencies and industry can generate immense quantities of data that would be particularly useful in microbial traceback and outbreak investigations, if shared publicly. There are currently several prohibitive factors that are slowing down broad implementation of these methods in food industries. These include sequencing costs and the lack of sufficient bioinformatics knowledge to analyze and interpret large sequencing data sets. However, sequencing is expected to become more affordable once sample preparation methods are optimized to better enrich microbial DNA, and bioinformatics training is becoming more accessible, making it easier to implement metagenomic methods.

## FOODBORNE PATHOGEN CONTROL: LEARNING FROM HEALTHY MICROBIAL ECOLOGIES

The majority of studies that have characterized microbiomes in food production or processing facilities have studied their association with food quality (14) or sanitation (15). However, there is still a significant gap in understanding of microbial ecologies associated with the presence and persistence of foodborne pathogens in the food processing environment. Furthermore, we know little about factors that differentiate microbial ecologies of contaminated facilities from those that are considered pathogen-free or “healthy.” My lab is studying associations of built food processing environment microbiota and the occurrence of foodborne pathogens. Our goal is to leverage this knowledge to develop microbiome-based pathogen control strategies that may be implemented in the food industry. Our study models include produce processing facilities where Listeria monocytogenes represents a high level of food safety concern. *L. monocytogenes* can adapt to harsh conditions encountered in food processing environments and persist in such environments over long periods. L. monocytogenes is widespread in natural environments and is commonly introduced into produce processing facilities with raw produce. Reduction of L. monocytogenes levels and its elimination from food processing environments are therefore critical in preventing its transmission to food.

Despite considerable efforts, control of L. monocytogenes has proven to be a challenging task, particularly in food processing facilities with suboptimal sanitary design of food processing equipment and also in the surrounding built environment, which can create niches suitable for environmental biofilm growth (16). Pathogens enclosed in a microbiome biofilm matrix that acts as a biophysical barrier protective against cleaners and sanitizers are more likely to persist in the environment (16). In cases of biofilm-supported pathogen persistence, chemical interventions frequently fail. Hence, enhancement of chemical cleaning and sanitation with biological pathogen control interventions using beneficial microorganisms (i.e., biocontrol strains) that improve the microbiological safety of the food processing environment may be a more effective alternative. Ideally, the biocontrol strains would be isolated from a similar food processing environment to be able to adapt and persist after introduction in new food processing environments. Biocontrol interventions have been successfully tested in meat processing facilities (14) but are yet to be evaluated in fresh produce processing environments, which would also need to take potential effects on produce quality into consideration. The advantages of biocontrol microorganisms include their ability to access difficult-to-clean niches in the food processing facility and to naturally compete with pathogens for available nutrients or inhibit their growth through the production of secondary metabolites.

The industry is becoming interested in advanced sanitation approaches based on understanding of microbial ecologies in the built food processing environments. Establishment of the Alliance for Advanced Sanitation, which connects industry with academia, is an important step toward formation of collaborations between academia and industry to tackle some of these issues. While there are many potential benefits to using biocontrol strains in food processing environments (especially those that are difficult to clean), they should complement rather than replace chemical and physical cleaning and sanitation practices to ensure that hygienic conditions are maintained in the food processing environment.

## CONCLUSIONS

Successful implementation of whole-genome sequencing in food safety has driven remarkable advances in foodborne pathogen tracking and surveillance. The next step toward advancing food safety is utilizing the vast amounts of available foodborne pathogen genomic sequences to extract functional information and identify biomarkers predictive of isolates’ pathogenic potential and other phenotypes relevant to food safety. Functional prediction will be critical for the optimization of detection methods and food safety risk assessment on a subspecies level that can have positive impacts on public health and the economic viability of food industries. Furthermore, going forward, we need to take a step beyond whole-genome sequencing and test the performance of metagenomic sequencing of food and clinical samples to facilitate identification of unknown causative agents of the vast majority of foodborne illness cases. Lastly, it is becoming increasingly important to understand the entire microbial landscape found in food systems to pinpoint the interactions and metabolic roles of individuals comprising microbial communities. This knowledge is necessary for the development of informed and biological pathogen control strategies. I anticipate establishment of metagenomic sequencing approaches in the food safety space with significant impact in the years ahead.
